# An Astaxanthin-Binding Ependymin-Related Purple Protein Responsible for the Coloration of a Marine Purple Sponge, *Haliclona* sp.

**DOI:** 10.3390/md23110441

**Published:** 2025-11-16

**Authors:** Takayuki Kaneko, Tomomi Asano, Shinji Kawasaki

**Affiliations:** Department of Molecular Microbiology, Tokyo University of Agriculture, 1-1-1 Sakuragaoka, Setagaya-ku, Tokyo 156-8502, Japan

**Keywords:** marine sponge, carotenoid, carotenoprotein, glycoprotein, ependymin, body coloration

## Abstract

Although marine sponges display strikingly diverse colors, the molecular basis of this color diversity remains largely unknown. Recently, the blue coloration of *Haliclona* sp. was attributed to a water-soluble carotenoprotein that binds orange astaxanthin (AXT) and mytiloxanthin (MXT) and belongs to the ependymin superfamily. Here, we investigated the coloration mechanism of a purple sponge, *Haliclona* sp. The purified purple protein was identified as a secreted glycoprotein, representing the second example of a color protein belonging to the ependymin superfamily. The blue and purple proteins were accordingly designated carotenoependymin (Cep)-Blue1 and Cep-Purple1. Cep-Blue1 binds orange AXT and MXT in a 1:1 ratio, whereas Cep-Purple1 binds only AXT, producing a smaller red shift than Cep-Blue1 in the 550–750 nm range. In vitro reconstitution of carotenoid-free apoproteins with their native carotenoids reproduced the original spectra. When the carotenoids bound to Cep-Blue1 and Cep-Purple1 were exchanged and reconstituted in vitro, Cep-Blue1 reconstituted with AXT exhibited a purplish-blue color, whereas Cep-Purple1 reconstituted with an equimolar mixture of AXT and MXT showed a preference for AXT and displayed an incomplete red shift. These results suggest that the subtle color variations among *Haliclona* species are determined by both species-specific carotenoid composition and the structural features of carotenoependymin proteins.

## 1. Introduction

Marine sponges are among the most colorful animals in the marine environment, exhibiting a wide range of hues even among closely related species. This diversity in coloration reflects the variety of pigments, highlighting these organisms as a prolific source of bioactive compounds. Pigments isolated from sponges include carotenoids [1,2], alkaloids [3,4], and melanins [5,6]; however, their specific contributions to body coloration, as well as the molecular mechanisms underlying subtle color variations, remain largely unknown.

Carotenoids are hydrophobic pigments typically displaying yellow to orange hues, and serve multiple roles, including coloration, antioxidation, and aposematic signaling [7,8]. In Porifera, they occur in free forms, such as β-carotene, zeaxanthin, and astaxanthin (AXT) [9,10]. Since animals cannot synthesize carotenoids de novo, sponges must acquire them from dietary sources, such as phytoplankton and microorganisms [2,10].

During our search for novel water-soluble carotenoproteins in microalgae to determine their distribution, function, and potential applications [11,12], we identified uncharacterized water-soluble carotenoproteins in marine sponges [13]. To date, five carotenoproteins have been reported in Porifera [13,14,15,16], including a water-soluble blue carotenoid-binding protein, EPD-BCP1 (renamed in this study as carotenoependymin-Blue1, Cep-Blue1), which was recently isolated from a blue sponge collected in Okinawa, Japan [13]. Cep-Blue1 is a secreted glycoprotein that induces a large bathochromic shift, converting orange carotenoids to blue and thereby contributing to the sponge’s blue coloration. However, only Cep-Blue1 has been characterized at the molecular level, including the determination of its primary structure.

Among marine carotenoproteins responsible for blue body coloration, only two have been structurally characterized: crustacyanin from crustaceans [17] and Cep-Blue1 [13]. Crustacyanin binds two molecules of AXT, whereas Cep-Blue1 binds one molecule each of structurally distinct AXT and mytiloxanthin (MXT) in a 1:1 ratio. Although these proteins belong to different protein families, both position their carotenoids at a heterodimer interface, a likely convergent evolution for producing a strong bathochromic shift.

Cep-Blue1 belongs to the ependymin superfamily, whose members are widely distributed across diverse organisms. While some ependymins have been implicated in processes such as memory consolidation [18,19] and optic nerve regeneration [20], the functions of most homologs remain unknown. As Cep-Blue1 is the only known ependymin protein linked to body coloration, the present study investigates whether related proteins contribute to other sponge colors. Here, a purple water-soluble ependymin-related carotenoprotein from a marine purple sponge is identified and characterized, and the molecular basis of the color difference between blue and purple in sponges is examined.

## 2. Results

### 2.1. Characterization of Sponge Samples

**Figure 1:**
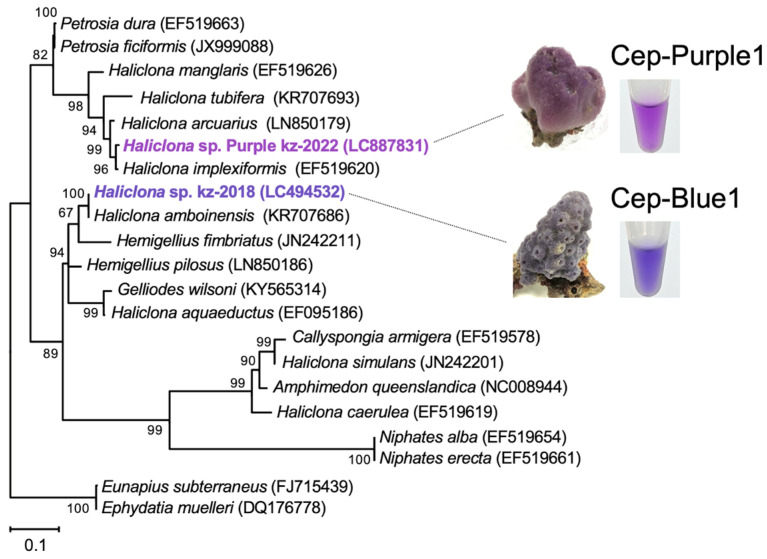
Phylogenetic tree based on *cox1* gene sequences, including purple and blue *Haliclona* sp. The tree was constructed using the maximum likelihood method based on the *cox1* gene sequences described in a previous study [13]. Numbers at the nodes indicate bootstrap values obtained using MEGA-X, and bootstrap proportions greater than 50% are shown next to the branches. The tree is rooted in the freshwater haplosclerids *E*. *subterraneus* and *E*. *muelleri*. GenBank accession numbers are indicated. Bar, 0.1 substitutions per nucleotide position.

The marine purple sponges used in this study were collected from coral reefs off the coast of Okinawa, Japan (Figure 1). Taxonomic characterization of the samples was performed based on morphology [21,22] and by sequencing the cytochrome *c* oxidase subunit 1 (*cox1*) [23,24] and the 18S rRNA gene [25]. The *cox1* sequences showed homology with validated Porifera species, including *Haliclona implexiformis* (accession no.: EF519620, 98% identity) [24] and *Haliclona arcuarius* (accession no.: LN850179, 96% identity) (Figure 1) [26]. Similarly, the 18S rRNA gene sequences showed homology with *Haliclona oculata* (accession no.: DQ927307, 97% identity) [27] and *Haliclona tubifera* (accession no.: KC902356, 97% identity) [25]. Each purple sponge exhibited morphological traits characteristic of the genus *Haliclona*, including spicule morphotypes, ladder-like choanosomal skeleton, and unispicular secondary line (Supplementary Figure S1) [21,22]. Based on both morphological and phylogenetic data, the sponge samples were identified as *Haliclona* sp. (Figure 1 and Figure S1).

### 2.2. Purification and Determination of Protein and Pigments

**Figure 2:**
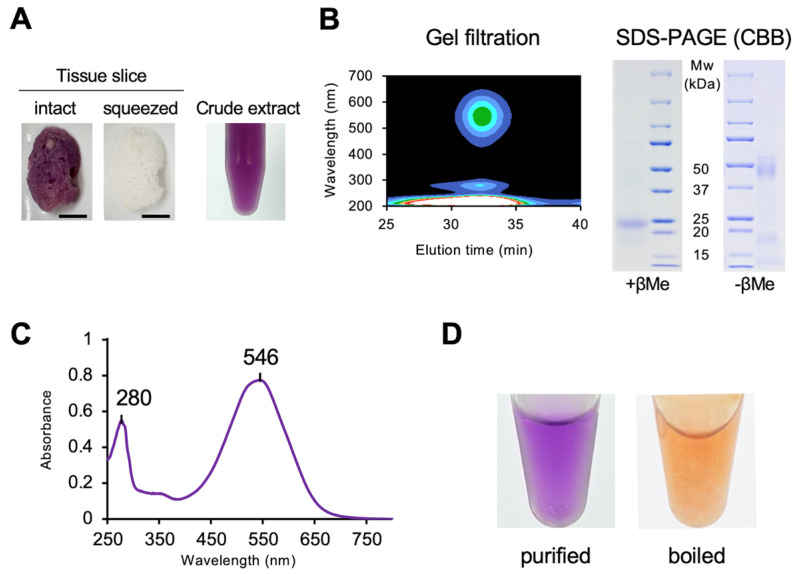
Purification of purple protein from a purple sponge, *Haliclona* sp.: (**A**) Change in body color before (**left panel**) and after (**right panel**) squeezing crude extracts from the sponge body. Scale bar, 1.0 cm. (**B**) Single elution peak detected with a photodiode array detector following gel filtration (**left panel**), and SDS-PAGE of the purified protein (**right panel**). (**C**) Absorption spectrum of the purified purple protein in Tris–HCl buffer (pH 7.5). (**D**) Color of the purified protein before (**left panel**) and after boiling (**right panel**).

The purple extract was obtained by manually squeezing the sponge body. The color of the freshly squeezed extract was identical to that of the sponge body, which turned faint brown after squeezing out the extract (Figure 2A). The purple supernatant was subjected to gel-filtration chromatography, yielding a single peak corresponding to the purple fraction (Figure 2B). The peak fraction was collected and further purified on a DEAE-Sepharose column, and the resulting purple fraction showed a single band with an apparent molecular mass of 23 kDa on SDS-PAGE (Figure 2B). Under non-reducing conditions, the purified protein migrated as a broad band of approximately 44–50 kDa (Figure 2B). Based on the retention time in HPLC size-exclusion chromatography, the apparent molecular mass of the native protein was estimated to be 41–43 kDa (Supplementary Figure S2), indicating that the purple protein is a dimer. The absorption spectrum of the purified protein exhibited maxima at 280 nm and 546 nm (Figure 2C). The native protein showed purple, whereas the boiled protein appeared orange (Figure 2D), indicating that the purple protein binds orange pigments and induces a bathochromic shift.

**Figure 3:**
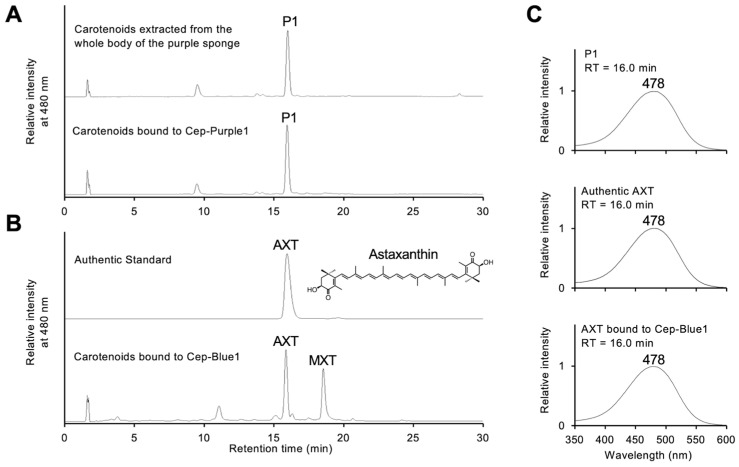
Carotenoid determination of the purified purple protein: (**A**) HPLC elution profiles of pigments extracted from the whole purple sponge body (**upper panel**) and those bound to purified Cep-Purple1 (**lower panel**). (**B**) HPLC elution profiles of authentic astaxanthin (**upper panel**) and pigments bound to Cep-Blue1 (**lower panel**). (**C**) Absorption spectra of peak P1 (**upper panel**), authentic astaxanthin (**middle panel**), and astaxanthin bound to purified Cep-Blue1 (**lower panel**). HPLC retention times (RTs) are indicated above the spectra, and the top wavelength of each peak is shown. The measured *m*/*z* values of AXT and P1 coincided, as described in the text. AXT, astaxanthin; MXT, mytiloxanthin.

The orange organic phase was extracted from the purple protein using the Bligh–Dyer method [13,28]. HPLC analysis of this phase on a C_18_ reversed-phase column yielded a single peak, designated P1 (Figure 3A). The retention time of P1 matched that of both authentic AXT and AXT bound to Cep-Blue1 (Figure 3A,B) [13]. Peak P1 shows a broad absorption maximum at 478 nm (Figure 3C). LC-MS analysis gave a predicted molecular formula of C_40_H_52_O_4_ ([M + H]+ at *m*/*z* = 597.3931, error = −0.0007). These results identified AXT as the carotenoid bound to the purified purple protein.

### 2.3. Primary Structure of the Purple Protein

**Figure 4:**
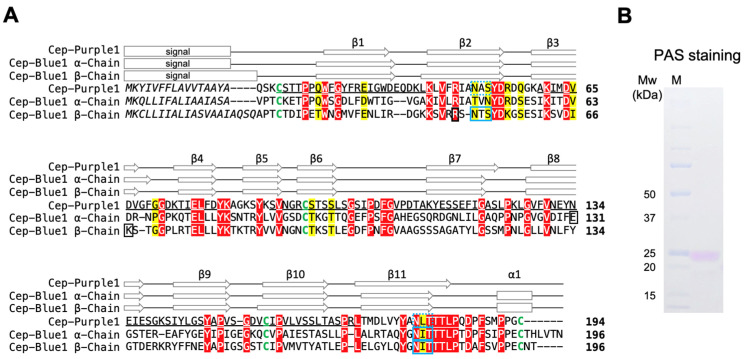
Primary structure of the purified purple protein compared with previously determined Cep-Blue1: (**A**) Alignment of the deduced amino acid sequences of Cep-Purple1 (accession no.: LC887830) and Cep-Blue1α and β chain (accession no.: LC494533 and LC737963). Sequences were aligned using ClustalW. Matched peptides by PMF analysis of Cep-Purple1 are underlined. Conserved signature motifs are indicated in yellow, and invariant residues are shown in red. The predicted (Cep-Purple1) or confirmed (Cep-Blue1) N-terminal signal sequences are shown in italic. Putative and confirmed N-glycosylation sites are indicated by dashed and solid blue boxes, respectively. Four conserved cysteine residues are shown in green. The three amino acid residues, including Arg49(β), that form hydrogen bonds with AXT in Cep-Blue1 are indicated by black boxes. Predicted (Cep-Purple1) or X-ray based (Cep-Blue1 α and β) secondary structures are indicated by open arrows (β-strands) and boxes (α-helices), respectively. The cleavage sites of the N-terminal signal sequence, N-glycosylation sites, and secondary structure of Cep-Blue1 were confirmed in a previous study based on crystallographic data. The secondary structure of Cep-Purple1 was predicted using Phyre2.2. (**B**) PAS staining of purified purple protein to detect protein glycosylation. The purified Cep-Purple1 is shown along with its corresponding molecular mass (kDa). PAS, periodic acid–Schiff.

Since N-terminal amino acid sequencing of the purple protein was not possible, probably due to N-terminal modification, peptide mass fingerprint (PMF) analysis against the cDNA library was conducted using an LC-MS/MS. A cDNA sequence that encodes purple protein was found in the cDNA sequence data from the purple sponge cDNA library, and a full-length cDNA was subsequently obtained by PCR (Figure 4A). The deduced amino acid sequence contained a putative EPD domain (accession no.: pfam00811), identified by BLAST search. The top blastp hit was a protein of unknown function from the demosponge *Amphimedon queenslandica* (accession no.: XP_003389285, 45% identity) [29], followed by Cep-Blue1 from *Haliclona* sp. (α-subunit, accession no.: 8I34_A, 34% identity; β-subunit, accession no.: 8I34_B, 34% identity) [13]. Although Cep-Blue1 is a heterodimer composed of α and β subunits, PMF analysis of the purified purple protein yielded a match to only one cDNA, implying that the purified protein is a homodimer.

Due to the limited availability of sponge samples, X-ray crystallographic studies of Cep-Purple1 have not yet been successful. Therefore, its primary and secondary structures were predicted computationally. Although the amino acid sequence identity with Cep-Blue1 was relatively low (34%), the four cysteine residues commonly conserved among EPD family proteins, the number and positions of α-helical and β-strand elements, and other key amino acid residues were nearly identical to those of Cep-Blue1 and other EPD family proteins (Figure 4A). The N-glycosylation sites confirmed in Cep-Blue1 by crystallographic analysis were also conserved in this protein. Periodic acid–Schiff staining further indicated that the purified protein was glycosylated (Figure 4B). Based on its carotenoid-binding property and sequence homology, the protein was designated as Cep-Purple1 (carotenoependymin-Purple1), an ependymin-related purple carotenoprotein.

### 2.4. Reconstitution Assays of Carotenoependymin Proteins

**Figure 5:**
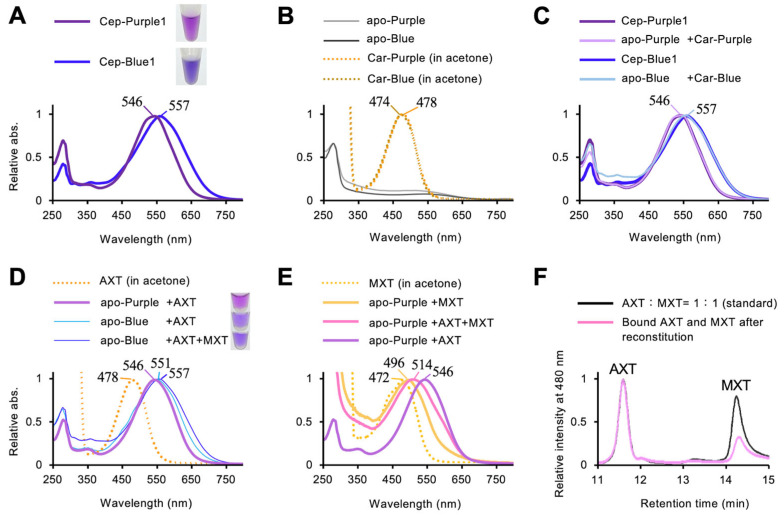
Reconstitution experiments using Cep-Purple1 and Cep-Blue1. Apo-Cep-Purple1 and apo-Cep-Blue1 are abbreviated as apo-Purple and apo-Blue, respectively. Carotenoids detached from Cep-Purple1 and Cep-Blue1 are denoted as Car-Purple and Car-Blue, respectively. The absorption maximum (λmax) of each spectrum is indicated: (**A**) Absorption spectra of purified Cep-Purple1 (purple line) and Cep-Blue1 (blue line). (**B**) Absorption spectra of apo-Purple (gray line), apo-Blue (black line), and their detached carotenoids. Car-Purple (orange dot line) and Car-Blue (brown dot line) were dissolved in acetone. (**C**) Absorption spectra of holo-Cep-Purple1 (pale purple line) and holo-Cep-Blue1 (pale blue line) reconstituted with their detached carotenoids, compared with native Cep-Purple1 (purple line) and Cep-Blue1 (blue line). (**D**) Absorption spectra of holo-Cep-Purple1 reconstituted with AXT alone (purple line), and holo-Cep-Blue1 reconstituted with AXT alone (pale blue line) or with an equimolar mixture of AXT and MXT (blue line). AXT in acetone is shown (orange dot line). (**E**) Absorption spectra of holo-Cep-Purple1 reconstituted with MXT alone (yellow line), an equimolar mixture of AXT and MXT (pink line), or AXT alone (purple line). MXT in acetone is shown (orange dot line). (**F**) HPLC elution profiles of the equimolar mixture of AXT and MXT used for reconstitution of apo-Cep-Purple1 (black line), and of the carotenoids bound after reconstitution (pink line).

Comparison of the absorption spectra of carotenoependymin proteins revealed a difference in the shoulder region, specifically a red shift between 550 and 750 nm, which appears to underlie their respective purple and blue hues (Figure 5A). To determine whether this shift results from differences in protein primary structures or carotenoid compositions, reconstitution experiments were performed. The absorption spectra of carotenoids detached from Cep-Purple1 and Cep-Blue1 were nearly identical in acetone, irrespective of the presence of MXT, which binds only to Cep-Blue1 (Figure 5B). Carotenoid-free apoproteins (apo-Cep-Purple1 and apo-Cep-Blue1) were colorless (Figure 5B) and were reconstituted with their respective detached carotenoids, referred to as Car-Purple and Car-Blue. In a previous study, apo-Cep-Blue1 fully recovered its native absorption spectrum upon reconstitution with its detached carotenoids [13]. Similarly, apo-Cep-Purple1 recovered its spectrum upon reconstitution with AXT (Figure 5C).

When apo-Cep-Blue1 was reconstituted with AXT alone, it failed to fully recover its native spectrum and exhibited a purplish-blue color (Figure 5D). When apo-Cep-Purple1 was reconstituted with MXT alone or with an equimolar mixture of AXT and MXT, it exhibited an incomplete red shift (Figure 5E). The binding ratio of carotenoids in reconstituted apo-Cep-Purple1 was estimated to be AXT:MXT = 3:1 (Figure 5F). These results indicate that Cep-Purple1 preferentially binds AXT.

## 3. Discussion

The genus *Haliclona* comprises diverse species that display a broad spectrum of colors, ranging across the entire visible spectrum from purple to red [22]. In this study, we purified a purple carotenoprotein responsible for the body coloration of a purple sponge, *Haliclona* sp., and identified it as a homolog of Cep-Blue1 belonging to the same ependymin superfamily. Although relatively few orthologs have been found in public databases for marine sponges, genes encoding EPD homologs are present in the genome of the marine sponge *Amphimedon queenslandica* [29]. *A. queenslandica* belongs to the family Chalinidae, which includes *Haliclona* species and exhibits diverse body colors [30]. Further studies may uncover novel EPD-related carotenoproteins not only in *Haliclona* but also in taxonomically distant sponge lineages.

This study suggests that the color difference between the blue and purple forms of *Haliclona* sp. originates from both the carotenoid composition and the protein structure. Cep-Purple1 binds only AXT, implying that the blue color of Cep-Blue1 results from the binding of AXT together with MXT. Reconstitution of Cep-Blue1 with AXT alone produced a purplish-blue color, indicating that the presence of MXT is important for generating the large red shift observed in Cep-Blue1. This result also highlights the importance of not only the carotenoid composition but also the protein structure, as a 5 nm red shift of the absorption peak was observed for AXT-bound Cep-Blue1 compared with AXT-bound Cep-Purple1 (Figure 5D). In contrast, reconstitution of Cep-Purple1 with MXT did not produce a large red shift (Figure 5E), suggesting that although MXT can bind, the binding pocket of Cep-Purple1 is structurally incompatible with MXT.

The carotenoid-binding preferences of Cep-Blue1 and Cep-Purple1 remain an important subject for future investigation. The binding carotenoid compositions, AXT and MXT in a 1:1 ratio for Cep-Blue1, and only AXT for Cep-Purple1, closely match the carotenoid compositions extracted from whole sponge bodies (Figure 3A) [13]. These results suggest that sponges acquire specific carotenoids to produce their characteristic coloration. However, the mechanisms underlying the acquisition of specific carotenoids remain largely unknown. Since sponges acquire nutrients through filter feeding and absorb various carotenoids from diverse environmental sources such as microorganisms and phytoplankton, it is unlikely that they selectively ingest only microorganisms containing AXT or MXT. Therefore, unknown mechanisms must be involved in the selective acquisition of preferred carotenoids or in the enzymatic conversion of carotenoid molecules within sponge cells. One possible candidate is the involvement of carotenoependymin proteins. Ependymin-related proteins are secreted glycoproteins localized on the cell surface, and some members are implicated in cell adhesion [31]. Water-soluble carotenoid-binding proteins, including carotenoependymin proteins, microalgal AstaPs of the fasciclin superfamily [11,12,32], and locust β-carotene-binding proteins of the Takeout superfamily [33,34], share common structural features such as a signal sequence for cell surface secretion and glycosylation, despite their distinct evolutionary origins. Each of these proteins binds a specific carotenoid. Regarding the AXT-binding site in carotenoependymin proteins, only Arg49 among the three amino acid residues responsible for forming hydrogen bonds with the β-end ring of AXT in Cep-Blue1 is conserved in Cep-Purple1 (Figure 4A) [13]. Although Arg49 may contribute to the binding preference for AXT, further discussion is limited due to the lack of structural data.

Among the carotenoproteins contributing to body coloration in marine animals, only two crystal structures have been determined to date: crustacyanin in crustaceans [17] and Cep-Blue1 [13]. Their heterodimerization, the molecular mechanisms underlying the AXT or AXT/MXT binding sites, and the mechanisms responsible for the associated red shift, including the conformational change in AXT from *6*/*6′-s-cis* to *6*/*6′-s-trans*, which contributes to the blue coloration of crustacyanin and Cep-Blue1, could only be elucidated through structural analyses. Future structural studies of Cep-Purple1 by X-ray crystallography or NMR spectroscopy, in comparison with other AXT-binding water-soluble carotenoproteins such as crustacyanin, Cep-Blue1, and microalgal AstaPs, are expected to clarify these mechanisms and the subtle color differences between blue and purple *Haliclona* species.

## 4. Materials and Methods

### 4.1. Animal Materials and Sponge Characterization

Three purple sponge specimens were collected between 2022 and 2023 from coral reefs off the coast of Okinawa, Japan, with the assistance of local fishermen. The collection of marine purple sponges is permitted in Japan, and this study did not involve any protected or endangered species. The use of marine sponges did not require ethical approval, as research on lower-level invertebrates is not restricted. We minimized the number of sponge specimens used in this study. The sponge samples were classified based on both morphological and genotypic characteristics. Fresh sponge specimens were fixed in 95% ethanol for morphological observation of spicule types, skeletal formations, and overall architecture. Skeletal structures and scleral features were examined under an optical microscope (Nikon, Tokyo, Japan). The morphological characteristics of the sponge samples were compared with those described for the genus *Haliclona* in “Systema Porifera: A Guide to the Classification of Sponges” [21,22]. Genomic DNA was extracted from sponge tissues using a DNeasy Tissue Kit (Qiagen, Hilden, Germany) according to the manufacturer’s protocol. The *cox1* gene was amplified by PCR using primers LCO1490 (5′-GGTCAACAAATCATAAAGATATTGG) and HCO2198 (5′-TAAACTTCAGGGTGACCAAAAAATCA) [23,24]. The 18S rRNA gene was amplified by PCR using primers SP18aF (5′-CCTGCCAGTAGTCATATGCTT) and SP18gR (5′-CCTTGTTACGACTTTTACTTCCTC) [25]. PCR products were sequenced by Macrogen (Seoul, Republic of Korea). The sequences of the *cox1* (657 bp) and 18S rRNA (1861 bp) genes were obtained from sponge samples. These sequences, along with those of taxonomically related species, were aligned using ClustalW 2.0 for phylogenetic analysis [35]. Phylogenetic trees were constructed using the maximum-likelihood methods with MEGA version 10.2.6 [36].

### 4.2. Purification and Characterization of the Purified Purple Protein

Fresh sponge samples were gently squeezed, and the released purple droplets were collected as crude extracts. Approximately 1 mL of purple extract (A_546_ = 2.0) was obtained from 1 g of sponge tissue after removal of excess seawater. Aqueous supernatants were obtained by ultracentrifugation at 100,000× *g* for 2 h. A single aqueous purple fraction was collected by passing through a gel-filtration column (HR100; GE Healthcare, Piscataway, NJ, USA) and DEAE Sepharose fast flow column (GE Healthcare, Piscataway, NJ, USA) using 50 mM Tris–HCl buffer, pH 7.5. Final purification yields of the purple protein ranged from 50 to 70%. After the purple fractions were collected, the concentrated proteins were checked for purity by SDS-PAGE. The molecular weights of the proteins were estimated using a commercial marker kit (Precision Plus Protein; Bio-Rad, Berkeley, CA, USA). Periodic acid–Schiff staining was performed using a commercial staining kit (Merck, Darmstadt, Germany) in accordance with the manufacturer’s instructions. The molecular mass of the native Cep-Purple1 was estimated by HPLC size exclusion chromatography (TSKgel G3000SWXL, TOSOH, Tokyo, Japan) and calibrated with the following molecular standards (Pharmacia, Uppsala, Sweden): ribonuclease (13.7 kDa), carbonic anhydrase (29 kDa), ovalbumin (44 kDa), conalbumin (75 kDa), aldolase (158 kDa), and blue dextran (2000 kDa).

### 4.3. cDNA Library Construction, Cloning, and Primary Structure Determination of Cep-Purple1

Total RNA was extracted with Trizol reagent (ThermoFisher Scientific, Bremen, Germany) and reverse transcribed into cDNA, which was used to generate a full-length cDNA library with the SMARTer Pico PCR cDNA Synthesis Kit (Takara Bio, Shiga, Japan) according to the manufacturer’s instructions. The cDNA library was sequenced using an Illumina NovaSeq 6000 Sequencing System (Illumina, San Diego, CA, USA). FASTQ files were assembled de novo using Trinity-v2.8.4 [37]. Approximately 54,000 contigs were generated after the de novo assembly and translated with TransDecoder-v5.5.0 (http://transdecoder.github.io (accessed on 13 November 2025)). To confirm the nucleotide sequence of cDNA encoding Cep-Purple1, PCR amplification was performed using the cDNA library as a template. PMF analysis was performed as previously described [12]. A piece of purified protein SDS-PAGE gel was excised, digested by trypsin (Promega). Peptide masses were determined in the positive-ion reflector mode in an Orbitrap Q Exactive focus LC-MS/MS system (ThermoFisher Scientific, Bremen, Germany) using a CAPCELL PAK C18 ACR reversed-phase column (150 × 2.0 mm i.d., 5 µm particle size, Shiseido, Tokyo, Japan). The solvent system was a mixture of water/formic acid (99.9/0.1, *v*/*v*, solvent A) and a mixture of acetonitrile/formic acid (99.9/0.1, *v*/*v*, solvent B). Positive ion mass spectra were recorded in full scan mode (*m*/*z* 200–2000) with an electrospray ionization (ESI) source. The ESI source parameters were set as follows: sheath gas flow rate of 10 units; capillary temperature of 320 °C; spray voltage of 4.0 kV; and S tube lens RF level of 50. Protein identification was performed using PEAKS Studio v8.5 (Bioinformatics Solutions Inc., Waterloo, ON, Canada) based on the cDNA libraries. The N-terminal amino acid sequencing was performed as previously described [13].

### 4.4. Extraction and Identification of Carotenoids

Carotenoids bound to the purified carotenoprotein were extracted using the Bligh-Dyer method [28]. The extracted carotenoids were completely dissolved in acetone. Total carotenoids from the whole sponge tissues were directly extracted with acetone. Carotenoid identification was performed as previously described [12,13]. Pigments bound to Cep-Purple1 were identified based on their absorption spectra, HPLC retention times, and molecular masses determined by high-resolution LC-MS/MS analysis, and were compared with those of Cep-Blue1 [13]. Astaxanthin was also obtained from Wako Chemicals (Osaka, Japan). Their absorption spectra and HPLC retention times were determined using an HPLC photodiode array system (200–700 nm) (L-2455; Hitachi, Tokyo, Japan) equipped with a CAPCELL PAK C18 MG ΙΙ reversed-phase column (150 × 4.6 mm i.d., 5 μm particle size; Shiseido, Tokyo, Japan). The solvent system was a mixture of methanol/water (80/20, *v*/*v*, solvent A) and a mixture of acetone/methanol (1/1, *v*/*v*, solvent B). Positive ion mass spectra were recorded in full scan mode (*m*/*z* 100–1500) with the same LC–MS/MS system and ESI source parameters. The data of molecular masses in high-resolution LC–MS/MS were analyzed with Compound Discoverer, version 3.3 software (ThermoFisher Scientific, Bremen, Germany) [12,13].

### 4.5. Preparation of Apoprotein and Its Reconstitution with Carotenoids

The reconstitution assay was performed as previously described [13,38]. Apoproteins were obtained by treating the purified protein with organic solvents: diethyl ether and acetone (1:1). AXT and MXT were detached from purified Cep-Blue1 and collected by C_18_-HPLC. Apoproteins were used for reconstitution assays after removing the organic solvents under an N_2_ gas stream. Apoprotein (dissolved in 50 mM Tris–HCl buffer [pH 7.5]) and a small amount of carotenoid solutions (dissolved in acetone) were mixed and incubated overnight on ice. Unbound insoluble carotenoids were removed by centrifugation at 15,000× *g* for 5 min, and the trace amounts of free carotenoids were removed by brief extraction with diethyl ether followed by removal of residual diethyl ether under a stream of N_2_ gas. Absorption spectra of apo- and holo-Cep proteins were measured using a spectrophotometer (UV-1800; Shimadzu, Kyoto, Japan).

### 4.6. Bioinformatics

Bioinformatic analyses were performed as previously described [13]. Briefly, database searches for sequence homology were performed using the programs blastp (http://www.ncbi.nlm.nih.gov/BLAST/, accessed on 15 October 2025) and FASTA (http://www.genome.jp/tools/fasta/, accessed on 15 October 2025) with standard parameters. The N-terminal signal sequence was predicted using SignalP 6.0 (https://services.healthtech.dtu.dk/services/SignalP-6.0/, accessed on 15 October 2025). The N-linked glycosylation sites were predicted using the program NetNGlyc 1.0 (https://services.healthtech.dtu.dk/services/NetNGlyc-1.0/, accessed on 15 October 2025). Protein sequences were aligned using ClustalW 2.0 [35]. Secondary structure predictions of Cep-Purple1 were performed using Phyre2.2 (https://www.sbg.bio.ic.ac.uk/~phyre2/html/page.cgi?id=index, accessed on 15 October 2025) [39]. The molecular mass was calculated by GENETYX-MAC software version 5.1 (Genetyx Corporation, Tokyo, Japan).

## Data Availability

Accession numbers and source data are presented in the main text and Supplementary Materials. All data used to support the findings of this study have been included in the manuscript and Supplementary information or made available through the National Centre for Biotechnology Information (NCBI). The sponge proteins are available from the corresponding author upon reasonable request.

## Supplementary Materials

The following supporting information can be downloaded at: https://www.mdpi.com/article/10.3390/md23110441/s1, Figure S1: Morphological characterization of *Haliclona* sp. Purple kz-2022. Figure S2: Gel filtration chromatography for determining the molecular weight of purified Cep-Purple1.

## Abbreviations

The following abbreviations are used in this manuscript:

EPD	Ependymin
EPD-BCP1	EPD-related blue carotenoprotein-1
Cep-Purple1	Carotenoependymin-Purple1
AXT	Astaxanthin
MXT	Mytiloxanthin
COX1	Cytochrome oxidase c subunit 1
HPLC	High-performance liquid chromatography
SDS-PAGE	Sodium dodecyl sulfate-polyacrylamide gel electrophoresis
