# Supplementary material for: An Astaxanthin-Binding Ependymin-Related Purple Protein Responsible for the Coloration of a Marine Purple Sponge, Haliclona sp
Source: Mar Drugs. 2025 Nov 16;23(11):441. doi: 10.3390/md23110441 (PMC12654638; doi:10.3390/md23110441)
Supplement: Supplementary file 1 [file marinedrugs-23-00441-s001.zip › marinedrugs-3962399-supplementary.pdf]

Figure S1

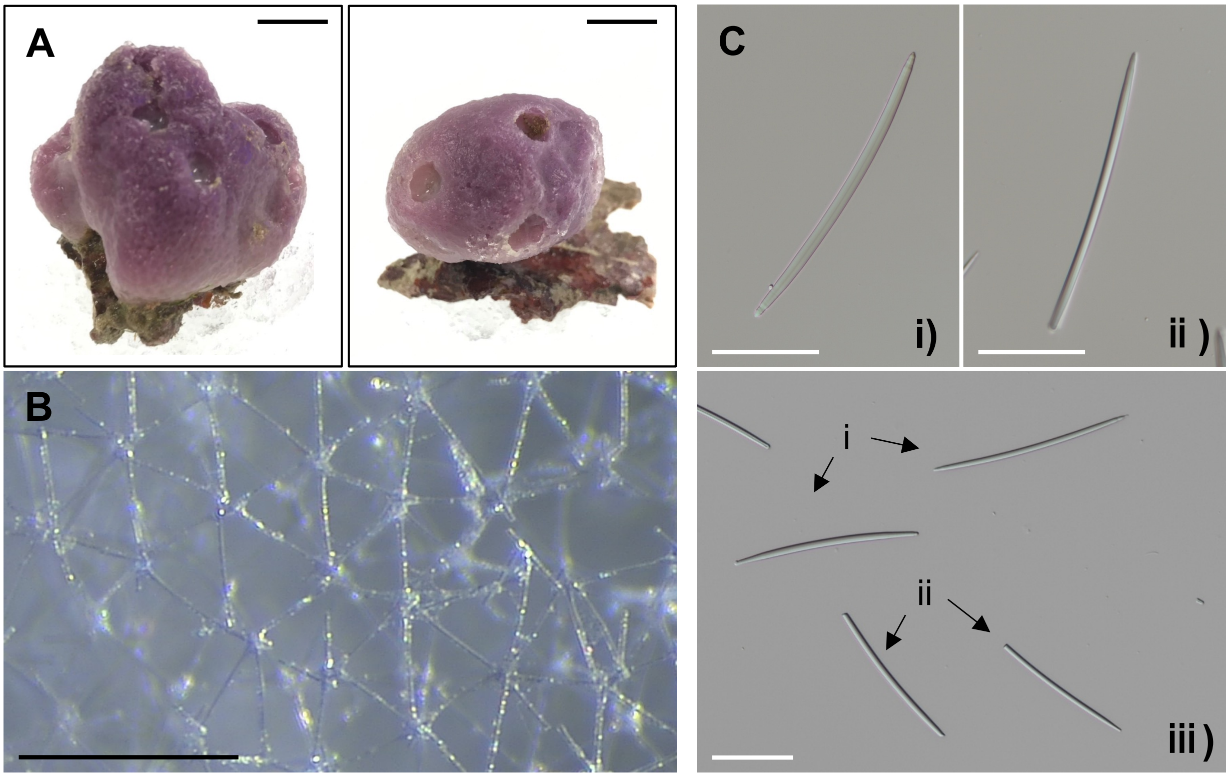

Figure S1. Morphological characterization of *Haliclona* sp. Purple kz-2022. (A) purple sponges used in this study, which were collected in 2022. Scale bar, 1.0 cm. (B) Perpendicular section of the choanosomal skeleton. Primary and secondary lines are regularly connected by unispicular fiber. Scale bar, 250 µm. *C,* spicule morphotypes. Oxeas are slightly curved, blunt or strongylote ends. i) oxeas type I, ii) oxeas type II. (iii) mixture of oxeas of different types. Scale bar, 50 µm.

Figure S2

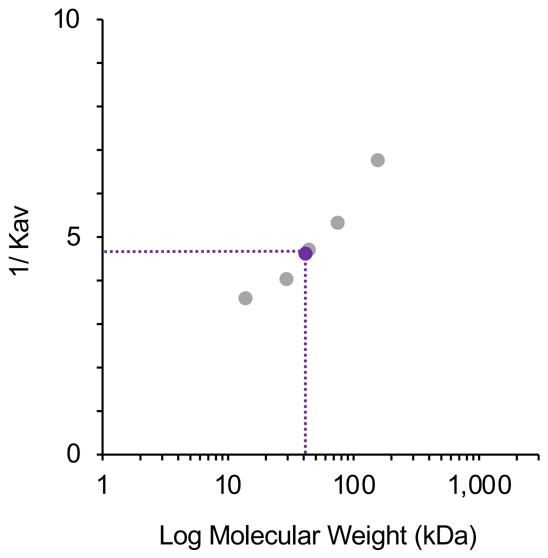

Figure S2. Gel filtration chromatography for determining the molecular weight of purified Cep-Purple1. The molecular weight of purified Cep-Purple1 was analyzed by HPLC size exclusion chromatography using a TSKgel G3000 SWXL column equipped with a photodiode array detector. Molecular mass standards (*gray plots*) included ribonuclease (13.7 kDa), carbonic anhydrase (29 kDa) , ovalbumin (44 kDa), conalbumin (75 kDa), and aldolase (158 kDa). The apparent molecular weight of Cep-Purple1 was estimated to be 41–43 kDa (*purple plot*).
